# Effects of Transcranial Alternating Current Stimulation on Repetitive Finger Movements in Healthy Humans

**DOI:** 10.1155/2018/4593095

**Published:** 2018-07-08

**Authors:** Andrea Guerra, Matteo Bologna, Giulia Paparella, Antonio Suppa, Donato Colella, Vincenzo Di Lazzaro, Peter Brown, Alfredo Berardelli

**Affiliations:** ^1^Neuromed Institute IRCCS, Pozzilli, Italy; ^2^Department of Human Neurosciences, Sapienza University of Rome, Rome, Italy; ^3^Unit of Neurology, Neurophysiology, Neurobiology, Department of Medicine, University Campus Bio-Medico, Rome, Italy; ^4^Nuffield Department of Clinical Neurosciences, John Radcliffe Hospital, University of Oxford, Oxford, UK; ^5^Medical Research Council Brain Network Dynamics Unit, Department of Pharmacology, University of Oxford, Mansfield Road, Oxford, UK

## Abstract

Transcranial alternating current stimulation (tACS) is a noninvasive neurophysiological technique that can entrain brain oscillations. Only few studies have investigated the effects of tACS on voluntary movements. We aimed to verify whether tACS, delivered over M1 at beta and gamma frequencies, has any effect on repetitive finger tapping as assessed by means of kinematic analysis. Eighteen healthy subjects were enrolled. Objective measurements of repetitive finger tapping were obtained by using a motion analysis system. M1 excitability was assessed by using single-pulse TMS and measuring the amplitude of motor-evoked potentials (MEPs). Movement kinematic measures and MEPs were collected during beta, gamma, and sham tACS and when the stimulation was off. Beta tACS led to an amplitude decrement (i.e., progressive reduction in amplitude) across the first ten movements of the motor sequence while gamma tACS had the opposite effect. The results did not reveal any significant effect of tACS on other movement parameters, nor any changes in MEPs. These findings demonstrate that tACS modulates finger tapping in a frequency-dependent manner with no concurrent changes in corticospinal excitability. The results suggest that cortical beta and gamma oscillations are involved in the motor control of repetitive finger movements.

## 1. Introduction

A growing number of studies on humans have shown that the two main natural rhythms of the primary motor cortex (M1), namely, beta (13–30 Hz) and gamma (30–100 Hz), play a role in motor control. Beta oscillatory activity increases during tonic contraction and decreases during movement preparation and execution [1–6]. By contrast, gamma oscillatory activity increases during movement preparation and execution [3, 7–9]. The contrasting functional effects of the two frequency bands of activity are supported by the effects of electrical stimulation on healthy subjects [10–12] and by the changes observed in patients with Parkinson's disease (PD). In this condition, untreated patients have elevated beta activity in basal ganglia-cortical circuits and slowed movement [13], whereas dyskinetic treated patients have elevated gamma activity at about 70 Hz and have excessive movement [14, 15].

Transcranial alternating current stimulation (tACS) is a recent noninvasive neurophysiological technique that entrains brain oscillations by inducing coherent changes in the firing and timing of populations of neurons [16]. The resulting neuronal synchronization may affect the activity of different cortical areas in a frequency-specific manner, resulting in the so-called “resonance principle.” Namely, the ability of tACS to modify brain rhythms especially when the externally superimposed oscillation is close to the natural frequency of the cortical area is stimulated [17, 18]. Accordingly, tACS can transiently entrain beta or gamma rhythms and modify the neuronal activity of M1 [19–22]. Beta and gamma tACS over M1 can also modulate voluntary movement performance in healthy subjects. Beta tACS delivered during a visually cued arm movement reduces movement velocity [10]. Similarly, the initial and peak force rates of the hand grip are both reduced when beta tACS is applied during a cued go/no-go task [11]. By contrast, gamma tACS improves initial and peak force generation in a hand grip task [11] and increases hand movement velocity and acceleration in a visually guided motor task [23].

Only one previous study has assessed the effects of tACS on fast finger tapping in healthy subjects [12]. Beta tACS was continuously delivered over M1 for 10 minutes, and motor performance was assessed 0, 30, and 60 minutes after stimulation was discontinued. The authors found that beta tACS slowed movement execution only at 0 min [12]. The effects of tACS were not assessed during the 10 minutes of stimulation. It is worth noting, however, that some effects of tACS delivered to M1 occur during, but not after, stimulation [21]. Moreover, not only did the study by Wach et al. not investigate the effects of gamma tACS, but the analysis of finger tapping was limited to tapping intervals used as an indirect measure of movement velocity and accuracy [12]. To investigate the effects of tACS on repetitive finger tapping is relevant for several reasons. First, repetitive finger movements are largely dependent upon M1 activation [24, 25], so we predict that these movements can be better modulated by noninvasive stimulation of cortical motor areas than proximal arm movements. Second, it is still not clear whether or not the oscillatory activity of M1 has a role in the generation of repetitive finger movements. Finally, repetitive finger tapping is one of the tests most commonly used in the clinical assessment of patients with parkinsonian syndromes. Namely, specific kinematic abnormalities of finger tapping, that is, the amplitude decrement (also known as the sequence effect) are hallmarks of PD [25–28]. Thus, a better knowledge of the effectiveness of tACS on motor control is essential for future studies in pathological conditions [29].

In the present study, we tested the effects of beta and gamma tACS on repetitive finger tapping. We performed a comprehensive kinematic analysis of various movement parameters (i.e., amplitude, velocity, and rhythm, as well as progressive amplitude and velocity changes associated with movement repetition) known to reflect different physiological mechanisms [26]. The results were also compared with those obtained during sham tACS. Lastly, in order to ascertain whether the effects of tACS on finger movements are due to concomitant changes in corticospinal excitability, we recorded motor-evoked potentials (MEPs) during tACS.

## 2. Material and Methods

### 2.1. Participants

The study enrolled 18 right-handed healthy subjects (7 females, age: 26.4 ± 3.5 (mean ± SD) years) with no history of neurological and psychiatric diseases or medication intake. None of the participants had any contraindications to non-invasive brain stimulation, as described in the latest international guidelines [30].

### 2.2. Motor Task and Kinematic Recordings

The motor task was adopted from previous studies [26–28, 31]. Repetitive finger movements were recorded using an optoelectronic motion system (Smart Motion System, BTS Engineering, Milan, Italy). This system comprises three infrared cameras (sampling rate, 120 Hz) that follow the 3D displacement of reflective markers taped to the participant's hand. We used five reflective markers (5 mm in diameter) of negligible weight. One marker was placed on the tip of the index finger, and another was put on the tip of the thumb. Three additional reflective markers were placed on the hand to define a reference plane that was used to mathematically exclude possible contamination due to unwanted hand movements from the index finger tapping recordings [26, 32].

To quantify repetitive finger movement kinematics, we used linear regression techniques to determine the intercept, which reflects the initial movement amplitude (degree) and initial velocity (degree/s), and the slope, which reflects the amplitude and velocity decrement during the movement repetition. Movement rhythm was also measured by the coefficient of variation (CV) of the intertap intervals (with higher values representing a lower regularity of repetitive movements). These analyses were performed on the first ten movements of the sequence as well as on the entire sequence, that is, 15 seconds of repetitive movements, as adopted in our previous study [26]. The focus on the initial movements was also motivated by the modified Movement Disorder Society UPDRS [33] which proposes a 10-tap trial in the assessment of finger tapping.

### 2.3. Brain Stimulation and Electromyographic Recordings

tACS was delivered through conductive rubber electrodes enclosed in saline-soaked sponges using BrainSTIM (EMS, Italy), with the stimulating electrodes (5 × 5 cm) placed over M1 and Pz, as detailed elsewhere [20]. Both the electrodes were secured in place using rubber strips around the head. The set-up was optimized in order to ensure that the impedance for stimulation, as measured by the stimulation device, would be <10 kΩ. tACS was delivered at two different frequencies: 20 Hz (beta) and 70 Hz (gamma). For gamma tACS, the frequency of 70 Hz was used as in previous studies investigating the effects of tACS on motor behaviour [11, 23]. Also, previous magnetoencephalographic studies in healthy subjects showed that the average peak frequency of movement-related gamma synchronization occurs at 70–75 Hz [9, 21, 34]. A sham tACS stimulation was used as a control. Similar to previous studies [23, 35], for sham tACS the stimulator was turned on only for 7 seconds (3 seconds of ramp-up, 1 second of stimulation, and 3 seconds of ramp-down). The frequency used for sham was 20 Hz, applied at the individual intensity used for beta tACS. Sine wave stimulation was delivered with no direct current offset and a peak-to-peak amplitude of 1 mA. If the participants complained unpleasant sensation (e.g., visual or skin discomfort), the stimulation intensity was lowered in steps of 0.05 mA until the discomfort was no longer perceived [11, 21, 35]. Thus, the mean stimulation intensity for beta tACS was 0.61 mA, while the intensity did not need to be adjusted for gamma tACS in any participant. This procedure ensured that subjects could also not be able to distinguish among the different stimulation conditions (including sham). Also, it allowed us to reasonably exclude the occurrence of any placebo or attentional effects due to perception of the stimulation.

Transcranial magnetic stimulation (TMS) was performed by using a MAGSTIM 200 (Magstim Company Limited, Whitland, South West Wales) connected to a standard figure-of-eight 70 mm coil delivering monophasic pulses. The TMS coil was held with the handle angled at 45° to the midsagittal line, pointing posteriorly and laterally. The precise area of cortical representation (“hotspot”) of the first dorsal interosseous (FDI) muscle of the right hand was targeted as the point from which stimuli at the minimal excitability threshold of TMS triggered MEPs of maximal amplitude and minimal latency in the target muscle. The MEPs were recorded through a pair of surface electrodes placed on the FDI muscle of the right hand in a belly/tendon montage. The resting motor threshold (RMT), that is, the stimulator's output able to elicit MEPs of ≥50 *μ*V peak-to-peak amplitude in at least 5 out of 10 consecutive stimuli, was determined, as was the minimum intensity needed to reliably produce MEPs of about 1 mV in size (MT1mV).

Electromyographic (EMG) signals were amplified by means of a Digitimer D360 amplifier (Digitimer Ltd., Welwyn Garden City, UK), digitized at 5 kHz (CED 1401 laboratory interface, Cambridge Electronic Design, Cambridge, UK), and stored on a laboratory computer for off-line analysis with dedicated software (Signal software version 5.08, Cambridge Electronic Design).

### 2.4. Experimental Design

**Figure 1:**
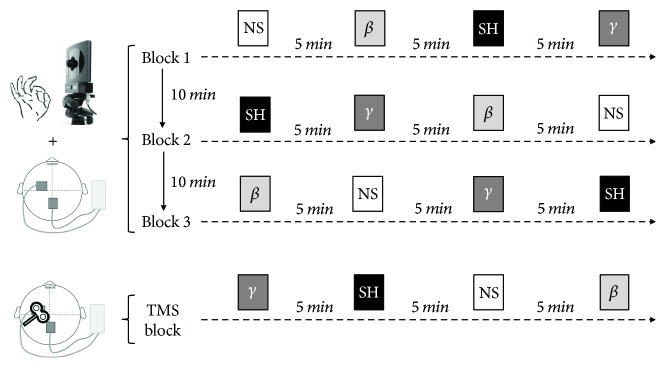
Experimental design. Finger tapping movements were recorded during no stimulation (NS), sham (SH), beta (*β*), and gamma (*γ*) tACS using an optoelectronic motion system. The four conditions were tested in a random order. We recorded one trial for each condition in 3 separate, consecutive blocks (total of 12 trials). Each trial consisted of 15 seconds of finger tapping. A 5-minute and 10-minute rest period was provided between each trial and between each block, respectively, to avoid fatigue. At the end of the kinematic recordings, the participants underwent a TMS assessment. Twenty single-pulse MEPs were recorded at rest during NS, SH, *β*, and *γ* tACS. The four conditions were randomized and performed at 5-minute intervals.

Participants were comfortably seated in a chair during the experimental procedures. We first applied single-pulse TMS before movement recordings in order to identify the FDI “hotspot” on the scalp. We then centered the tACS electrode on the FDI “hotspot” and recorded the finger tapping movements during tACS. Four conditions were tested in a random order: 20 Hz (beta tACS), 70 Hz (gamma tACS), sham tACS, and no stimulation (baseline). The motor task consisted of 12 trials in total. We recorded one trial for each condition in 3 separate, consecutive blocks. Each trial consisted of 15 seconds of finger tapping, performed at the maximal voluntary rate. During the motor task, the participants were continuously encouraged to tap with as large and fast movements as possible. A 5-minute and 10-minute rest period was provided between each trial and between each block, respectively, to avoid fatigue between trials and across blocks (Figure 1). Of note, the motor task started about 10 seconds after the beginning of the stimulation, so that for the sham condition, the motor task was performed while the stimulation was off. One practice trial was allowed before the kinematic recordings started to allow the subjects to become familiar with the experimental procedure.

The participants underwent a TMS assessment at the end of the kinematic recordings. The FDI “hotspot” targeting was repeated over the sponge of the stimulating electrode, and the site was marked with a felt-tip pen to allow the coil to be repositioned more easily during collection of the MEPs. The MT1mV was then determined. Twenty single-pulse MEPs were recorded at rest during beta, gamma, sham, and off tACS. The four conditions were randomized and performed at 5-minute intervals.

The participants were blinded to the stimulation conditions and unable to distinguish them. The experimenter who analyzed the kinematic measures and the MEPs was also blinded to the stimulation paradigm. There was only one operator who was not blinded to the experimental procedure, that is, the researcher who applied tACS and set up the stimulation frequencies.

### 2.5. Statistical Analysis

To evaluate the effects of tACS on finger tapping kinematics, we performed a repeated measures analysis of variance (ANOVA) using CONDITION (four levels: beta, gamma, sham, and baseline) and SEQUENCE (two levels: first ten movements and whole sequence) as within-subject factors. Different kinematic variables were analyzed in separate ANOVAs. To evaluate the effects of tACS on MEP amplitude, we performed a repeated measures analysis of variance (ANOVA) using CONDITION (four levels: beta, gamma, sham, and baseline) as the within-subject factor. Fisher's pairwise least significant difference test was used for post hoc analyses in ANOVAs.

Pearson's product-moment correlation was used to evaluate possible associations between the effects of tACS on movement kinematics and M1 excitability. For this purpose, we normalized the kinematic and TMS values recorded during beta and gamma tACS to their respective baselines (no stimulation).

Unless otherwise stated, all the results are shown as mean values ± standard error of the mean (SEM). The level of significance was set at *P* < 0.05 in all the tests. Data were analyzed using Statistica® (StatSoft Inc.).

## 3. Results

### 3.1. Effects of tACS on Finger Tapping Kinematics

**Figure 2:**
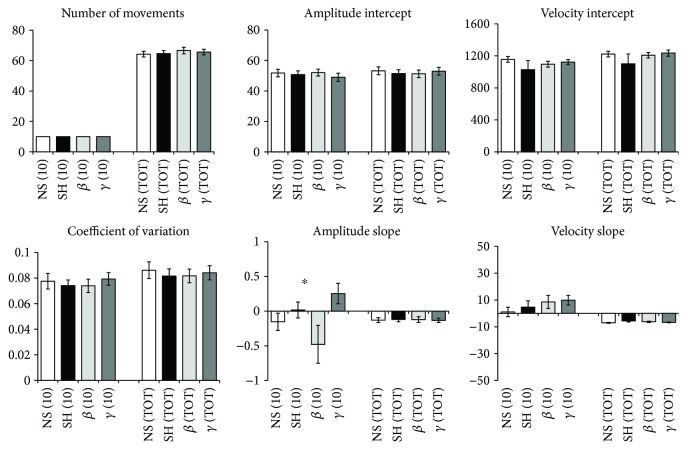
Kinematic variables. (10) refers to the first ten movements and (TOT) refers to the whole motor sequence. NS, SH, *β*, and *γ* refer to no stimulation, sham, beta, and gamma tACS, respectively. The asterisk denotes a significant CONDITION × SEQUENCE interaction in a repeated measures ANOVA. Error bars indicate the standard error of the mean.

The kinematic variables of repetitive finger tapping in the four tACS conditions are shown in Figure 2. Two-way repeated measures ANOVA revealed a significant effect of CONDITION for the amplitude decrement (*F*(3, 51) = 3.00, *P* = 0.03); the post hoc analysis revealed higher values during gamma tACS and lower values during beta tACS (*P* < 0.01). Most importantly, the analysis on the amplitude decrement detected a significant CONDITION × SEQUENCE interaction (*F*(3, 51) = 3.42, *P* = 0.02). The post hoc analysis showed that the effects of tACS occurred in the early phase, that is, during the first ten movements of the motor task, with higher values being observed during gamma tACS (*P* = 0.01) and lower values during beta tACS (*P* = 0.04) in comparison with those of the unstimulated baseline tapping performance. Moreover, there was no difference between sham tACS and baseline tapping performance (*P* = 0.31). In addition, the analysis of the amplitude decrement did not detect any effect of SEQUENCE (*F*(1, 17) = 0.13, *P* = 0.72). The analysis did not reveal any effect of CONDITION, for the other finger tapping kinematics (all *P* > 0.05).

The remaining results are represented in Table 1 and indicate the main effect of SEQUENCE on the slope and intercept of tapping velocity. The former was a consequence of a drop in velocity (negative velocity slope) across the whole trial (physiological fatigue) as opposed to a drop in velocity within the first 10 movements, over which there was a slight increase in velocity (Figure 2). The change in the intercept of tapping velocity between the first 10 movements and the whole series of movements was a product of the linear regression technique, given these small differences in slopes. Importantly, however, these effects were independent of stimulation.

Further analyzing our data, rather than estimating the intercepts and slopes, we calculated the average amplitude and velocity for the first 10 movements of the motor task and for the whole sequence of 15 seconds. These measures were analyzed by a repeated measures ANOVA using CONDITION (four levels: beta, gamma, sham, and baseline) and SEQUENCE (two levels: first ten movements and whole sequence) as within-subject factors. This analysis showed a significant effect of SEQUENCE for both parameters (average amplitude: *F*(1, 17) = 34.68, *P* < 0.001; average velocity: *F*(1, 17) = 6.82, *P* = 0.01). Post hoc analysis indicated higher values for both measures during the first ten movements (average amplitude: 46.57 ± 1.41°; average velocity: 438.62 ± 16.71°/sec) in comparison to those measured for the whole sequence (average amplitude: 43.88 ± 1.18°; average velocity: 418.43 ± 13.96°/sec). This analysis, however, showed no significant effect of CONDITION and no CONDITION × SEQUENCE interaction for both parameters (all *P* > 0.05). The results were therefore consistent with physiological fatigue across the whole trial, irrespective of the trial type.

Also, since tACS affected the slope of tap amplitudes over the first ten movements of the tapping task and yet velocity remained unchanged, we checked whether there were any changes in tapping frequency during tACS over the first ten movements. We calculated the average frequency and intercept and slope of the linear regression of the instantaneous tapping frequency versus the tap number over the first ten movements of the motor sequence. Then, we performed three separate repeated measures ANOVAs using CONDITION (four levels: beta, gamma, sham, and baseline) as the within-subject factor. The analyses showed no significant results (frequency average: *F*(3, 51) = 2.26, *P* = 0.09; frequency intercept: *F*(3, 51) = 0.65, *P* = 0.59; and frequency slope: *F*(3, 51) = 1.12, *P* = 0.35).

We finally explored whether fatigue might accrue during the experimental recordings or whether the rest periods provided between the four (intrablock) stimulation conditions and the three recording blocks (Figure 1) were sufficient to prevent this. We therefore assessed the occurrence of intrablock fatigue using a repeated measures ANOVA using CONDITION ORDER (four levels: 1st, 2nd, 3rd, and 4th condition) and SEQUENCE (two levels: first ten movements and whole sequence) as within-subject factors. We also assessed the occurrence of fatigue across consecutive blocks using a repeated measures ANOVA using BLOCK ORDER (three levels: 1st, 2nd, and 3rd block) and SEQUENCE (two levels: first ten movements and whole sequence) as within-subject factors. The two ANOVAs did not reveal any significant effects of the main factors of analysis, nor any significant interaction between them (all *P* > 0.05, lowest *P* = 0.12).

**Figure 3:**
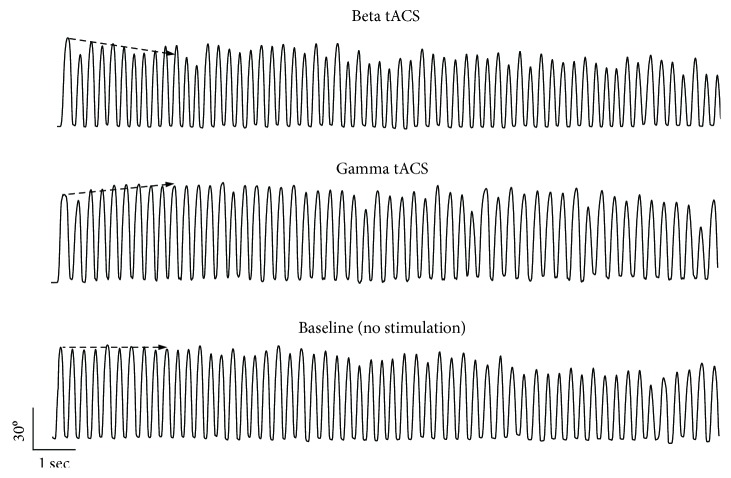
Paradigmatic example of kinematic recordings of finger tapping during beta and gamma tACS. The participant's performance was frequency-dependently modulated by tACS. Namely, beta tACS led to an early amplitude decrement during repetitive finger tapping while gamma tACS had the opposite effect.

In summary, the results indicate that the participants' performance was frequency-dependently modulated by tACS. Beta tACS led to an early amplitude decrement while gamma tACS had the opposite effect during the first ten movements of the motor sequence (Figure 3). Other measures of movement amplitude and velocity were similar in all four tACS conditions examined. Lastly, the results also provide evidence of longer-term physiological fatigue in terms of a drop in average amplitude and velocity across all taps when compared to the first 10 taps. However, this was unaffected by stimulation or stimulation condition order and did not carry over between blocks.

### 3.2. Effects of tACS on MEP

Relative MEP amplitude was not modulated by M1 tACS delivered at different frequencies (no stimulation/baseline: 0.97 ± 0.03; beta: 0.96 ± 0.04; gamma: 1.01 ± 0.05; and sham: 0.99 ± 0.04 mV). A repeated measures ANOVA on MEP values did not reveal any significant effect of the main factor CONDITION (*F*(3, 51) = 1.24, *P* = 0.30), thereby indicating that tACS delivered in different conditions did not modify M1 excitability. These findings suggest that tACS did not modulate corticospinal excitability at the current intensities used.

### 3.3. Intensity-Dependent Effects of Beta tACS

Since the stimulation intensity of beta tACS (0.61 ± 0.05 mA) was on average significantly lower than that of gamma tACS (1.00 ± 0.00 mA), we also investigated possible intensity-dependent effects of beta tACS on movement kinematics and MEP amplitude. We applied the median split procedure for this purpose [21]. We divided the participants into two groups according to the intensity of stimulation for beta tACS: a low-beta-intensity group (9 subjects, 0.37 ± 0.04 mA) and a high-beta-intensity group (9 subjects, 0.84 ± 0.05 mA). Kinematic data and MEP values during beta tACS were normalized to their corresponding baseline (no stimulation). We then conducted separate ANOVAs, using the between-group factor INTENSITY (two levels: high versus low beta) and the within-group factor SEQUENCE (two levels: first ten movements and whole sequence). The analysis on amplitude slope did not reveal any significant effects of INTENSITY (*F*(1, 16) = 0.20, *P* = 0.65) or SEQUENCE (*F*(1, 16) = 1.68, *P* = 0.21), nor any INTENSITY × SEQUENCE interaction (*F*(1, 16) = 0.35, *P* = 0.55). No significant effects were observed for the main factors and their interaction term for either of the other kinematic parameters (all *P* > 0.05). Similarly, beta tACS intensity did not have any effect on normalized MEP (high beta subgroup: 0.97 ± 0.04 mV versus low beta subgroup: 1.09 ± 0.07 mV (*P* = 0.12) by unpaired *t*-test). Lastly, we also investigated possible correlations between the stimulation intensity during beta tACS and the normalized amplitude slope and other kinematic variables (both during the first ten movements and during the whole sequence), none of which were found to be statistically significant (*r* values ranged between −0.01 and 0.20 and the *P* value was always >0.05). Similarly, no significant correlation emerged between the beta tACS intensity and the MEP amplitude ratio (*r* = −0.14; *P* = 0.57).

### 3.4. Correlations between Movement Kinematics and MEP

In this analysis, we aimed to verify whether the kinematic variable modulation of repetitive finger movements correlated, at the individual level, with the MEP amplitude recorded during beta and gamma tACS. We did not detect any correlation between kinematic variables during the first ten movements of the sequence and M1 excitability changes either during beta tACS (*r* values ranged between −0.25 and 0.19 and the *P* value was always >0.05) or during gamma tACS (*r* values ranged between −0.01 and 0.38 and the *P* values were always >0.05). Similarly, we did not detect any correlation between kinematic variables measured on the whole 15-second sequence and M1 excitability changes either during beta tACS (*r* values ranged between −0.12 and 0.19 and the *P* value was always >0.05) or during gamma tACS (*r* values ranged between −0.15 and 0.48 and the *P* values were always >0.05). These data further suggest that the frequency-dependent effects of tACS on movement kinematics were unrelated to individual M1 excitability changes.

## 4. Discussion

In the present study, we demonstrate that in healthy humans, beta tACS leads to an early and progressive reduction in amplitude (amplitude decrement) during repetitive finger tapping while gamma tACS has the opposite effect. These frequency-dependent stimulation effects are observed during the first ten movements of the motor sequence. As the tapping sequence continues still, further physiological fatigue sets in [23], but this is unaffected by tACS. tACS, as applied here, does not significantly affect other movement parameters, including overall movement amplitude, velocity, and rhythm which are mediated by distinct physiological mechanisms [26–28]. Finally, tACS does not induce any changes in MEP amplitude.

Since the amplitude, velocity, and rhythm of finger tapping in our study were similar in all four tACS conditions examined, the effects of beta and gamma tACS on the early amplitude decrement cannot be ascribed to varying levels of motor performance. Moreover, the changes in initial amplitude slope during beta and gamma tACS are unlikely to be due to any effect of physiological fatigue because the presentation of conditions was randomized and successive blocks were performed at least ten minutes apart. Accordingly, the effects of fatigue carrying over successive blocks were excluded by means of a specific statistical analysis on consecutive measurements. We can also rule out the possibility that the effects of beta and gamma tACS on the amplitude decrement are due to nonspecific factors (e.g., scalp or visual sensations) because we lowered the stimulation intensity of tACS to a level at which these effects were not present. We may thus speculate that the effects of tACS on the amplitude decrement result from an interaction between the modulation of motor resonant brain rhythms induced by stimulation and the physiological mechanisms involved in repetitive finger tapping. This would be in line with evidence that tACS at appropriate frequencies may entrain local oscillations, and, where these are the product of circuit resonances, amplify such rhythms [8–12].

Previous studies have shown that oscillatory activity in the beta frequency range varies with motor behaviour. In physiological conditions, beta activity is considered to have an antikinetic effect; that is, it is enhanced during movement suppression and depressed during voluntary movement execution [4, 36, 37]. The early but short-lived progressive amplitude decrement during repetitive finger tapping seen during beta tACS may be therefore explained by the entrainment of beta activity in M1, with or without amplitude amplification through resonance effects. In contrast, gamma tACS led to an early but short-lived progressive increment in tapping amplitude. Synchronized oscillations in the gamma frequency band are also functionally relevant in human M1. A rapid increase in the power of gamma oscillations occurs before and during movement execution as well as during rapid action stopping [2, 7, 38–41]. Gamma activity in M1 has been considered to be a prokinetic rhythm [42, 43] or to underlie flexible motor control [41]. Therefore, by entraining neuronal activity in the gamma frequency band, gamma tACS may exert prokinetic effects on repetitive finger movements and promote the dynamic control of motor output. Due to the limited topographical specificity of the electric stimulation, another possible explanation for prokinetic effects of gamma tACS on M1 is the concurrent modulation of the somatosensory cortex (S1). High gamma cortical activity is also a natural rhythm of S1 [44–47], and high gamma tACS on S1 is known to modulate central sensory processing [47]. Therefore, the improvement of motor performance at the beginning of the tapping sequence could be due to changes in sensory processing. A further possibility for the effects of tACS on repetitive finger movements is the modulation of frontal areas other than M1. Among these, the anterior cingulate cortex (ACC) has a role in planning and executing motor sequences [48, 49]. Entrainment of the gamma and beta rhythm might then facilitate or interfere with converging motor input from upstream areas, like the ACC, to M1. The fact that both the effects of beta and gamma tACS were only evident early on during movement sequences raises the possibility that their functional effects rapidly saturate. Indeed over time, they are compounded by the effects of physiological fatigue which overtake repetitive movement sequences irrespective of the stimulation state. Another possible explanation is the occurrence of a ceiling effect for gamma tACS and the activation of compensatory mechanisms counteracting the detrimental effect of beta tACS in physiological conditions.

The absence of tACS-related changes in movement velocity observed in our study is at odds with observations in previous studies [10, 11, 23], but we did not focus on repetitive finger movements and tested more proximal arm movements or alternative tasks, such as the hand force grip. In addition, while we evaluated internally generated voluntary movements, Pogosyan et al. [10], Joundi et al. [11], and Moisa et al. [23] tested externally cued motor tasks, which are known to be generated by different brain networks [50–52].

Another result of our study is that beta and gamma tACS did not modify the level of corticospinal excitability, as measured by changes in MEP amplitude following single-pulse TMS. This is in line with previous studies reporting similar findings in healthy subjects [20, 21, 35, 53], although other studies imply otherwise [19, 22, 54–56]. We have also found that there was no correlation between MEP change and early amplitude slope modulation during tACS on M1. This result suggests that modifications in cortical oscillations rather than changes in the global level of M1 excitability are responsible for the amplitude decrement of repetitive finger movements.

The present results may provide a background for future studies investigating voluntary movements in physiological conditions and movement abnormalities, like bradykinesia, in patients. The term bradykinesia is clinically defined as slowness and reduced amplitude of voluntary movement that is exacerbated by repetitive actions [57, 58]. Several studies have suggested that changes in the oscillatory activity in the basal ganglia or coupling between cortical and subcortical rhythms are all putative mechanisms involved in various hand movement abnormalities in PD patients [41, 59–63]. The observation that tACS delivered in the beta range leads to an early amplitude decrement may suggest that one of the mechanisms that underlies amplitude decrement in PD is an excess of beta oscillations in M1 and its connections.

The present study has a number of limitations that warrant consideration. Due to a lack of data allowing a direct estimation of tACS effects on brain oscillations, the interpretation of the mechanisms underlying our results remains speculative. Most importantly, the effects of tACS on movement kinematics may be relatively specific to finger control. It may not be possible to extrapolate the results we obtained in our study by testing repetitive finger movements to repetitive movements of other body segments, which may differ both in terms of inertia of the moving part and of the segment's underlying physiological mechanisms. In addition, despite the existing controversies on non-invasive electrical stimulation (e.g., topographical specificity and intensity-related effects) [64], several studies have demonstrated significant effects on the cortical areas being targeted [20, 21, 65] and no additional advantages in using stronger stimulation intensities once these reach about 1 mA [66]. In this regard, our results also demonstrate that the effects of stimulation, namely, beta tACS, are not influenced by the different intensities applied. Also, we did not use a navigation system for the TMS procedure. Finally, although we examined corticospinal excitability using single-pulse TMS, we cannot exclude that other physiological mechanisms known to be affected by beta and gamma tACS, such as cortical interneuronal excitability, [20, 21, 67] contributed to our results. The assessment of the possible relationship between tACS-dependent behavioural changes and concurrent changes of interneuronal activity is beyond the present study, and future studies are needed to explore this issue.

In conclusion, this study provides novel information on the effects of tACS, delivered at functionally relevant frequencies, on motor behaviour in healthy human subjects. Our findings point to a physiological role of cortical beta and gamma oscillations in the organisation and execution of repetitive finger movements. The novel finding of the study is the demonstration of differential effects of gamma and beta tACS on repetitive finger movements, specifically on the amplitude decrement. This could help in better understanding the role of cortical oscillations in the generation and modulation of sequence effect. The results also have pathophysiological implications and suggest that the amplitude decrement observed in PD may be due to exaggerated endogenous oscillatory activity in the beta band in M1 and its connections. The hope is that it may be possible to ameliorate movement abnormalities in PD through frequency-specific tACS, as already evidenced in tremor [29]. Further studies are needed to fully understand the behavioural effects of tACS in healthy subjects and in patients with movement disorders, as well as the clinical implications of tACS for therapeutic purposes in patients.

## Figures and Tables

**Table 1:** Effect of CONDITION (four levels: beta, gamma, sham, and baseline), SEQUENCE (two levels: first ten movements and whole sequence), and their interaction on movement kinematics. Significant effects are shown in bold. Post hoc tests confirmed that the main effect of CONDITION and the CONDITION × SEQUENCE interaction for amplitude slope was due to a frequency-dependent modulation of the amplitude slope estimated over the first 10 movements, but not over the whole trial. In contrast, the main effect of sequence on the slope and intercept of tapping velocity reflected a drop in velocity across the whole trial as opposed to a drop in velocity within the first 10 movements, over which there was a slight increase in velocity (Figure 2). Importantly, however, the longer-term physiological fatigue-related effects on velocity were independent of stimulation condition.

	CONDITION			SEQUENCE			CONDITION × SEQUENCE		
	*F*	*d*, *f*	*P*	*F*	*d*, *f*	*P*	*F*	*d*, *f*	*P*
N Movements^∗^	0.57	3.51	0.63	—	—	—	—	—	—
CV	0.66	3.51	0.57	2.64	1.17	0.12	0.17	3.51	0.91
Amplitude intercept	0.85	3.51	0.47	3.26	1.17	0.09	4.52	3.51	0.07
Velocity intercept	1.28	3.51	0.29	62.13	1.17	**<0.001**	1.43	3.51	0.24
Amplitude slope	3.00	3.51	**0.03**	0.13	1.17	0.72	3.42	3.51	**0.02**
Velocity slope	1.97	3.51	0.12	44.30	1.17	**<0.001**	0.89	3.51	0.45

^∗^shown are only the results of CONDITION, since the number of movements considered in the early part of the motor task is always 10.
